# Optical Diffraction in Close Proximity to Plane Apertures. III. Modified, Self-Consistent Theory

**DOI:** 10.6028/jres.109.033

**Published:** 2004-10-01

**Authors:** Klaus D. Mielenz

**Affiliations:** National Institute of Standards and Technology, Gaithersburg, MD 20899-8440

**Keywords:** bidirectional scalar fields, boundary-value theory, circular apertures, diffraction, Kirchhoff, irradiance, near zone, optics, polarization, Rayleigh, scalar wave functions, slits, Sommerfeld, transmission coefficients

## Abstract

The classical theory of diffraction at plane apertures illuminated by normally incident light is modified so that diffraction on the source side of the screen is taken into consideration and the energy transport across the aperture plane is described by continuous functions. The modified field expressions involve the sums and differences of the Rayleigh-Sommerfeld diffraction integrals as descriptors of a bidirectional flow of energy in the near zones on either side of the aperture. The theory is valid for unpolarized fields, and a pragmatic argument is presented that it is applicable to metallic as well as black screens. The modified field expressions are used for numerical near-field computations of the diffraction profiles and transmission coefficients of circular apertures and slits. In the mid zone the modified theory is reduced to the Fresnel approximation, and here the latter may be used with confidence.

## 1. Introduction

This is a continuation of previous papers [1,2] in which the physical significance of the classical Rayleigh-Sommerfeld and Kirchhoff diffraction integrals was assessed and their suitability for computations in the near zone was analyzed. The need for such computations arises, for example, in the evaluation of radiometric diffraction errors, where it is necessary to know the transmission coefficients of the apertures used for the measurements. The computation of these coefficients is a near-zone task even for large aperture-detector distances.

The specific situation considered is a plane aperture A contained in an infinitesimally thin screen S that occupies the *xy*-plane of a Cartesian coordinate system and is illuminated from the half space *z* < 0 by a normally incident monochromatic plane wave with irradiance *E*_0_ and wavelength *λ*. The resulting optical field is denoted by a scalar wave function,

$$U(P)=\sqrt{E_{0}}u(P),|u(P)|\leq 1,$$ (1)

and is expressed in the Rayleigh-Sommerfeld theory in terms of the surface integrals,

$$u_{\text{RS}}^{\left(p\right)}(P)=-\frac{ik}{2\pi }\int_{A}\text{dQ}\frac{e^{ikQP}}{QP},\;z>0,$$ (2a)

$$u_{\text{RS}}^{\left(s\right)}(P)=\frac{1}{2\pi }\int_{A}\text{dQ}\frac{\partial }{\partial z}(\frac{e^{ikQP}}{QP})=\frac{1}{ik}\frac{\partial u_{\text{RS}}^{\left(p\right)}}{\partial z},\;z>0$$ (2b)

where a metallic screen illuminated by *p*- or *s*-polarized light is assumed.^1^ The corresponding expression in Kirchhoff's theory, which is usually associated with black screens, is

$$\begin{array}{l} u_{K}(P)=-\frac{1}{4\pi }\int_{A}\text{dQ}[ik-\frac{\partial }{\partial z}]\frac{e^{ikQP}}{QP}\\ \equiv \frac{1}{2}[u_{\text{RS}}^{\left(p\right)}(P)+u_{\text{RS}}^{\left(s\right)}(P)],\;z>0. \end{array}$$ (2c)

In these equations, A denotes the aperture area, P = (*x*,*y*,*z*) is the point of observation, Q = (*ξ*,*η*,0) is a point inside the aperture, *QP* is the distance between these points, dQ is the surface element at Q, *k* = 2*π*/λ is the circular wave number, and the time dependence of the field is assumed as e^−i^*^ωt^*.

Equations (2a,b) were reduced in Ref. [1] to previously unknown single integrals for the respective cases of circular apertures and apertures bounded by straight lines, and these were used for numerical computations of $u_{\text{RS}}^{\left(p\right)}$ and $u_{\text{RS}}^{\left(s\right)}$ that involved no simplifying assumptions and could be performed for arbitrarily small distances *z* from these apertures. The numerical results obtained were everywhere finite, free of singularities, and confirmed the well-known prediction that $u_{\text{RS}}^{\left(p\right)}$ and $u_{\text{RS}}^{\left(s\right)}$ reproduce the boundary values assumed in their derivation ( $\partial u_{\text{RS}}^{\left(p\right)}\to ik$ and $u_{\text{RS}}^{\left(s\right)}\to 1$ as *z* → 0) but not the compatible values $u_{\text{RS}}^{\left(p\right)}\to 1$ and $u_{\text{RS}}^{\left(s\right)}/\partial z\to ik$) which are implied in the classical postulate that the aperture field is the same as the unperturbed geometrical field incident on the screen. These inconsistencies obscured the differences between the Rayleigh-Sommerfeld and Kirchhoff integrals in the immediate proximity of the screen and made it impossible to assess their physical significance without additional considerations.

This impasse was overcome in Ref. [2] by evaluating Eqs. (2a,b) for the special case of a diffracting half plane and comparing them to the corresponding values, $u_{S}^{\left(p\right)}$ and $u_{S}^{\left(s\right)}$, given by Sommerfeld’s rigorous theory of half-plane diffraction [3,4]. The agreement was remarkably good on the positive side of the screen, where the differences $\left(u_{\text{RS}}^{\left(p,s\right)}-u_{S}^{\left(p,s\right)}\right)$ and their derivatives were negligibly small even at sub-wavelength distances *z*. Thus, it was decided that the aperture values given by the Rayleigh-Sommerfeld integrals are consistent with Sommerfeld’s rigorous theory, so that attempting to improve them would be pointless.

Accordingly, it became apparent that the real problem with the Rayleigh-Sommerfeld and Kirchhoff theories was not their failure to reproduce the assumed boundary values but these boundary conditions themselves. The classical theories involve “inclination factors” which explicitly preclude a backward motion of diffracted light and, thus, any perturbation of the geometrical field on the source side. On the other hand, Sommerfeld’s rigorous theory showed that the incident light is modified by diffraction before it reaches the screen, and therefore the notion of an unperturbed incident field is abandoned in this paper by adding a diffraction term to the geometrical field on the source side.

The comparison with Sommerfeld’s theory also suggested the need for a further modification of the classical theory. The optical field specified by the rigorous theory is expressed in the form $u_{S}^{\left(p,s\right)}=u_{S}\pm {\hat{u}}_{S}$, where $u_{S}^{\left(p,s\right)}$ obey the same boundary conditions as the Rayleigh-Sommerfeld integrals and their components *u*_S_ and *û*_S_ propagate in the opposite directions of the incident field and its reflection from the screen and are mutually incoherent. In this paper, the Rayleigh-Sommerfeld integrals will likewise be resolved into forward and reverse components defined by

$$u_{K}=\frac{1}{2}(u_{\text{RS}}^{\left(p\right)}+u_{\text{RS}}^{\left(s\right)}),\;{\hat{u}}_{K}=\frac{1}{2}(u_{\text{RS}}^{\left(p\right)}-u_{\text{RS}}^{\left(s\right)}),\;z>0,$$ (3a)

where the subscript K is used because the forward wave function on the left-hand side of this equation happens to be the same as the Kirchhoff diffraction integral, Eq. (2c). The effective, time-averaged flow of field energy is then given by the squared moduli of these functions, so that the mutually incoherent, forward and reverse irradiances incident on any given area element d*x*d*y* are given by

$$E=E_{0}|u_{K}{|}^{2},\;\hat{E}=E_{0}|{\hat{u}}_{K}{|}^{2},\;z>0.$$ (3b)

Finally, these quantities will be extended into the source space by matching functions so that the overall field is continuously differentiable^2^ in the aperture plane and the bidirectional transport of energy through the aperture is also expressed by continuous functions. The modified theory presented in this paper is valid for normally incident light but can easily be adapted for oblique angles of incidence. As will be shown, it becomes indistinguishable from the usual Fresnel approximation in the mid zone *z* ≫ *λ*, and here the latter can be used with confidence.

## 2. Modified Field Expressions

### 2.1 Derivations

In addressing the problem of diffraction on the source side of a plane metallic screen illuminated by normally incident parallel light, it is frequently assumed that

$$\begin{array}{l} \nu^{\left(p,s\right)}=u_{+}^{\left(p,s\right)},\;z>0,\\ \;=e^{ikz}\pm e^{-ikz}\pm u_{-}^{\left(p,s\right)},\;z<0, \end{array}$$ (4a)

where *ν* ^(^*^p^*^,^*^s^*^)^ is the total field, $u_{\pm }^{\left(p,s\right)}$ denotes the field is the components due to diffraction, and e^i^*^kz^* ± e^−i^*^kz^* is the unperturbed geometrical field on the source side. These assumptions appeared first in Rayleigh’s papers [5,6] on diffraction by infinitesimally small apertures and show that a continuously differentiable solution for *ν*^(^*^p,s^*^)^ must obey the boundary conditions

$$u_{-}^{\left(p\right)}=2+u_{+}^{\left(p\right)},\;\frac{\partial u_{-}^{\left(p\right)}}{\partial z}=\frac{\partial u_{+}^{\left(p\right)}}{\partial z},\;z=0,$$ (4b)

$$u_{-}^{\left(s\right)}=u_{+}^{\left(s\right)},\;\frac{\partial u_{-}^{\left(s\right)}}{\partial z}=2ik+\frac{\partial u_{+}^{\left(s\right)}}{\partial z},\;z=0.$$ (4c)

These conditions were used by Rayleigh to derive the initial terms of Taylor expansions for $u_{\pm }^{\left(p,s\right)}$ for slits and circular apertures with dimensions smaller than the wavelength of light. Additional higher-order terms were calculated by Sommerfeld [4], Bouwkamp [7], and others.

As mentioned above, the Rayleigh-Sommerfeld integrals, Eq. (2a,b), will be retained in this paper by assuming

$$u_{+}^{\left(p,s\right)}=u_{\text{RS}}^{\left(p,s\right)}(x,y,z),\;z>0,$$ (5a)

and then the second condition in Eq. (4b) and the first condition in Eq. (4c) will be satisfied by also assuming

$$u_{-}^{\left(p\right)}=-u_{\text{RS}}^{\left(p\right)}(x,y,-z),\;u_{-}^{\left(s\right)}=u_{\text{RS}}^{\left(s\right)}(x,y,-z),\;z<0.$$ (5b)

However the two remaining conditions in Eqs. (4a,b) are still not satisfied, so that ∂*ν* ^(^*^p^*^)^/∂z and *ν* ^(^*^s^*^)^ will still be discontinuous in the aperture plane.

This failure of Eqs. (4a) can be attributed to the fact that the Rayleigh-Sommerfeld integrals are composite quantities which can be resolved into the forward and reverse field components *u*_K_ and *û*_K_ in Eqs. (3a); that is, $u_{\text{RS}}^{\left(p\right)}=u_{K}+{\hat{u}}_{K}$ and $u_{\text{RS}}^{\left(s\right)}=u_{K}-{\hat{u}}_{K}$. There are no physical reasons why these sums and differences should be continuously differentiable for *z* = 0, but on the other hand this must be required of *u*_K_ and *û*_K_ in order to correctly account for a continuous transport of energy through the aperture. To satisfy this requirement, we retain Eq. (5a) but reverse the signs of e^−i^*^kz^* and $u_{\text{RS}}^{\left(s\right)}$ in Eqs. (5b), so that

$$u_{-}^{\left(p\right)}=u_{\text{RS}}^{\left(p\right)}(x,y,-z),\;u_{-}^{\left(s\right)}=-u_{\text{RS}}^{\left(s\right)}(x,y,-z),\;z<0.$$ (6a)

Hence, by applying Eqs. (3a) and letting $\nu =\frac{1}{2}(\nu^{\left(p\right)}+\nu^{\left(s\right)})$, $\hat{\nu }=\frac{1}{2}(\nu^{\left(p\right)}-\nu^{\left(s\right)})$,

$$\begin{array}{l} \nu =u_{K}(x,y,z),\;z>0,\\ \;=e^{ikz}+{\hat{u}}_{K}(x,y,-z),\;z<0, \end{array}$$ (6b)

$$\begin{array}{c} \hat{\nu }=u_{K}(x,y,z),\;z>0,\\ \;=-e^{\text{-i}kz}+u_{K}(x,y,-z),\;z<0. \end{array}$$ (6c)

Now, it may be recalled that the Rayleigh-Sommerfeld integrals obey the boundary conditions assumed in their derivation; that is,

$$u_{\text{RS}}^{\left(s\right)}\equiv ik\frac{\partial u_{\text{RS}}^{\left(p\right)}}{\partial z}=1\;\text{in}\;A,\;\equiv 0\;\text{on S,}\;z\to 0,$$ (6d)

and hence it follows at once that the scalar field specified by Eqs. (6b,c) is continuously differentiable in the aperture plane. Likewise, the corresponding forward and reverse irradiances,

$$\begin{array}{l} E=|u_{K}(x,y,z){|}^{2},\;z>0,\\ \;=|e^{ikz}+{\hat{u}}_{K}(x,y,-z){|}^{2},\;z<0, \end{array}$$ (7a)

$$\begin{array}{l} \hat{E}=|{\hat{u}}_{K}(x,y,z){|}^{2},\;z>0,\\ \;=|-e^{ikz}+u_{K}(x,y,-z){|}^{2},\;z>0, \end{array}$$ (7b)

are continuously differentiable, thus implying a smooth bidirectional transport of energy through the aperture.

Equations (6b,c) and (7a,b) represent the key findings of this paper. It should be noted that in these expressions the roles of *u*_K_ and *û*_K_ are reversed on opposite sides of the screen. That is, *û*_K_ appears in the expressions for the forward field quantities *u*_K_ and *E*, and vice versa. The general properties of these modified field quantities can readily be predicted from the results reported in Ref. [1]; namely, that the differences between the Rayleigh-Sommerfeld integrals $u_{\text{RS}}^{\left(p\right)}$ and $u_{\text{RS}}^{\left(s\right)}$ are pronounced only in the immediate proximity of the screen and vanish in the Fresnel approximation. Thus, a bidirectional exchange of energy between the positive and negative sides of the aperture occurs only in the near zone, long as *û*_K_(*x*,*y*,|*z*|) is appreciably different from zero for values of |*z*| on the order of a few wavelengths. In the Fresnel limit on either side of the aperture plane (|*z* | ≫ *λ*) the forward and reverse fields are unidirectional, and the forward field is reduced to the standard expressions in terms of Fresnel integrals for slits and Lommel functions for circular apertures [8] for *z* > 0, and to the unperturbed geometrical field for *z* < 0. Similarly, the reverse Fresnel field is zero for *z* > 0 and equal to a Fresnel diffraction pattern superimposed on the reflected geometrical field for *z* < 0.

### 2.2 Numerical Examples

#### 2.21 Slits

**Fig. 1:**
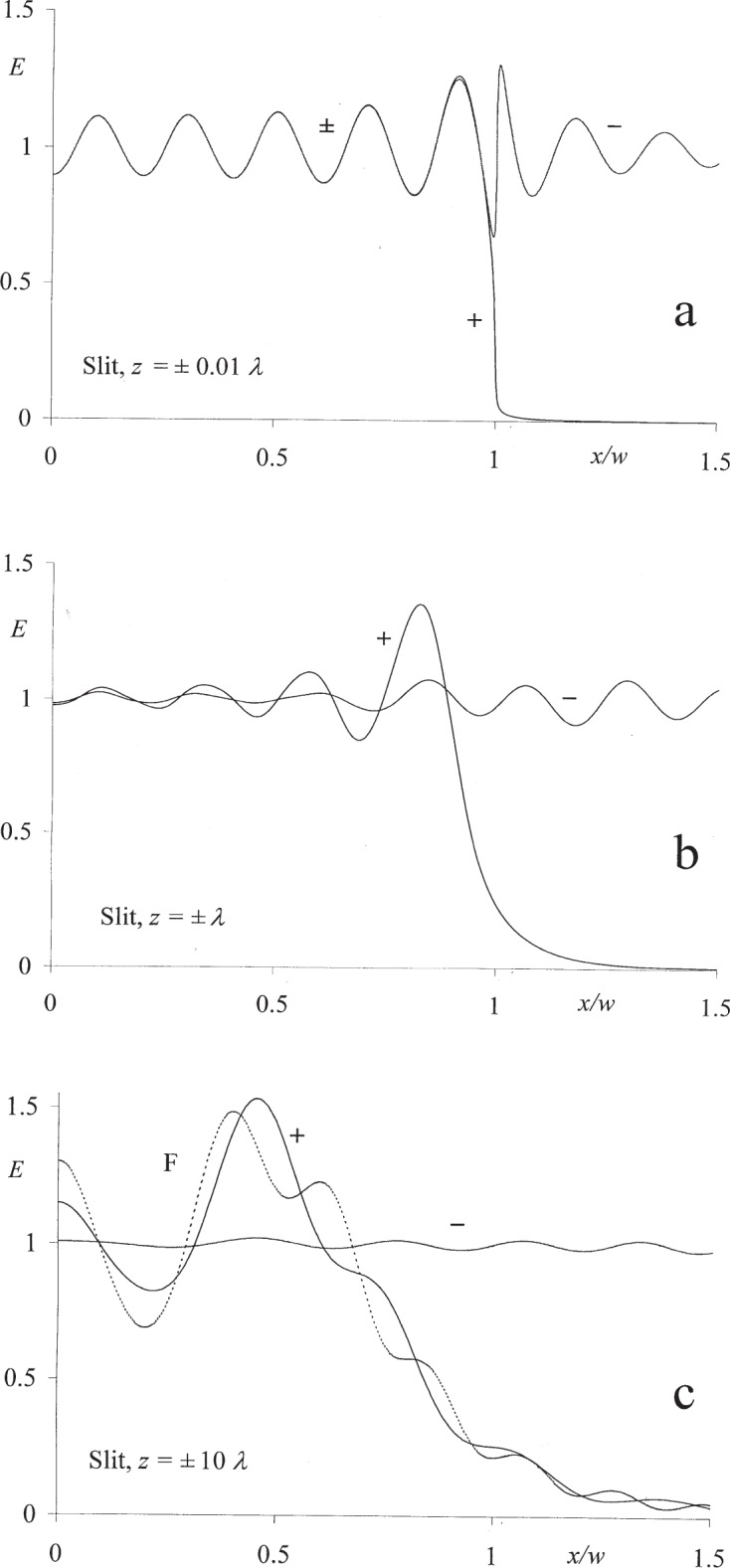
Forward irradiance profiles on opposite sides of the aperture plane for a slit of width 2 *w* = 10 *λ*. (a) *z* = ± 0.01 *λ*, (b) *z* = ± *λ*, (c) *z* = ± 10 *λ*, F = Fresnel approximation.

As an illustration of the behavior of the modified field expression on both sides of the aperture plane, Figs. 1a–c show the forward irradiance profiles [*E*(*x*,*z*) vs *x*/*w*]^3^ for a slit of width 2 *w* = 10 *λ* and for varying distances ± *z* from the aperture plane. The numerical values shown in these figures were computed using Eqs. (3a) and (7a) in conjunction with the expressions for $u_{\text{RS}}^{\left(p,s\right)}$ derived in Sec. 3.3 of Ref. [1].

Figure 1a shows that, for *z* = ± 0.01 *λ*, the modified field irradiances are manifestly continuous inside the aperture (*x*/*w* < 1) and that a modulation of the incident field by diffraction also occurs on the opaque portion of the screen (*x*/*w* < 1). For *z* = ± *λ*, shown in Fig. 1b, the diffraction profile on the positive side of the screen is already significantly altered in that more light is spreading into the shadow, whereas on the negative side the modulation of the field is diminished. Finally, for *z* = ± 10 *λ* as shown in Fig. 1c, the profile on the positive side is similar but not yet equal to the Fresnel approximation (F, shown as a dashed line) and the modulation of the incident field on the negative side is very small. This confirms the expectation that the modified theory affects only the positive and negative near zones in which the Fresnel approximation does not apply. For the slit width assumed here, it is estimated that the Fresnel limit is reached, within 1 % or better, for |z| = 100 *λ*.

### 2.22 Circular Apertures

**Fig. 2:**
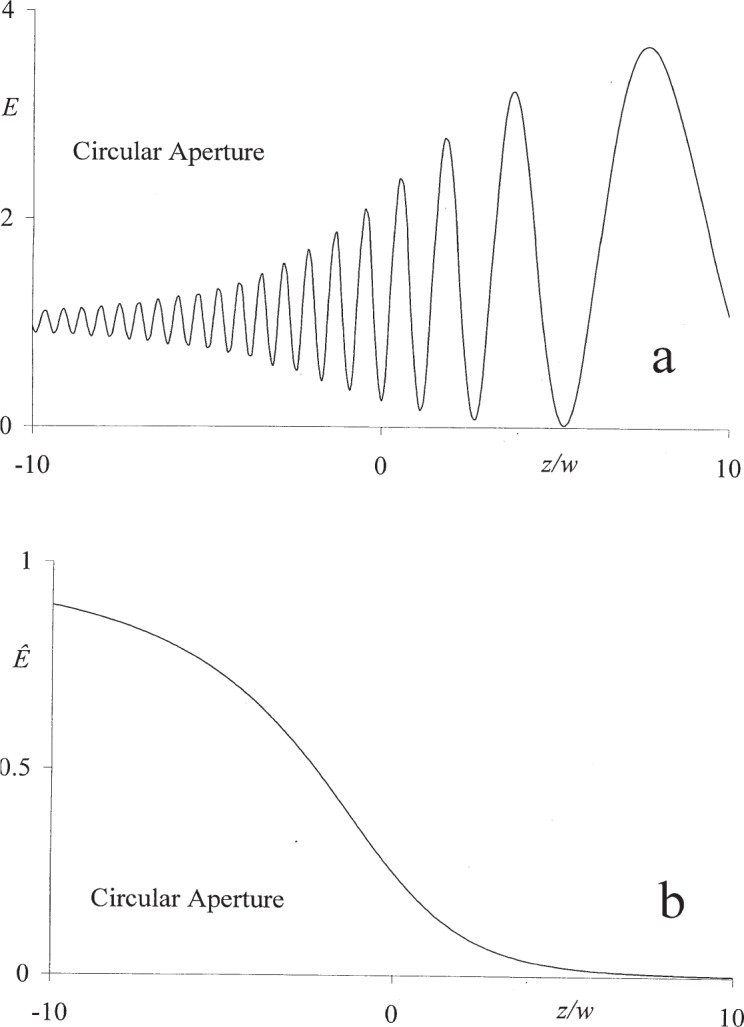
Variation of axial irradiance on opposite sides of the aperture for a circular aperture of diameter 2 *w* = 10 *λ*. (a) Forward irradiance *E*, (b) reverse irradiance *Ê*.

**Fig. 3:**
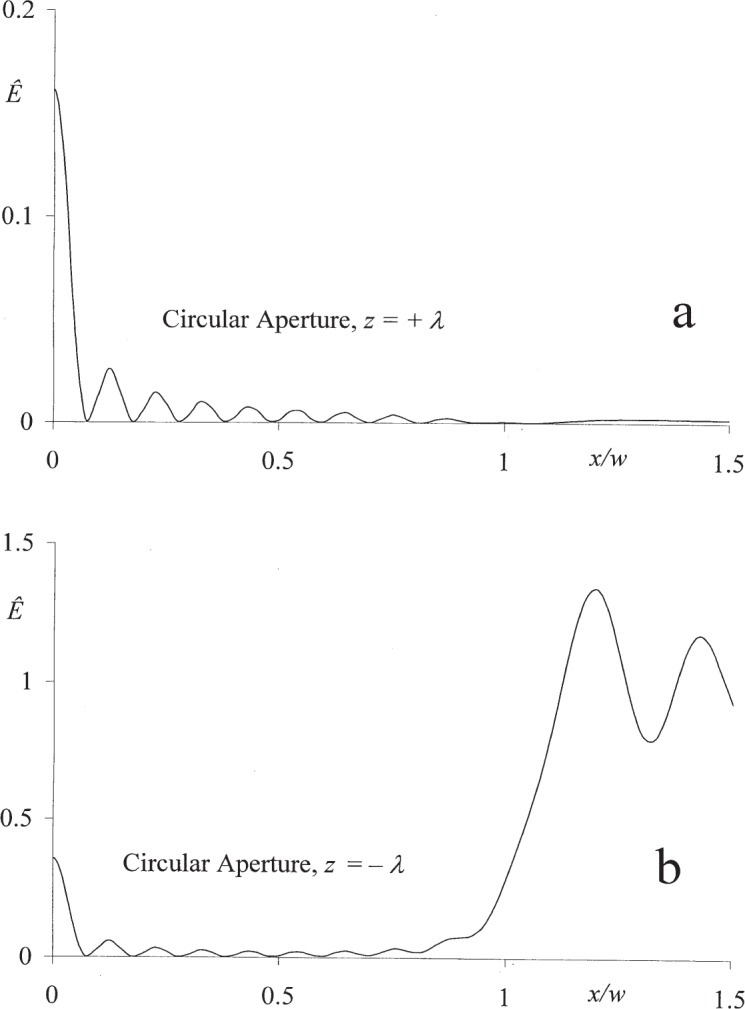
Reverse irradiance profiles on opposite sides of the aperture plane for a circular aperture of diameter 2 *w* = 10 *λ*. (a) *z* = *λ*, (b) *z* = −*λ*.

The numerical data presented in Figs. 2 and 3 illustrate the bidirectionality of the field in the positive and negative near zones of a circular aperture of width 2 *w* = 10 *λ*. The data were computed using the mathematical expressions derived in Sec. 3.2 of Ref. [1].

Figures 2a,b show the forward and reverse axial irradiances *E*(0,*z*) and *Ê*(0,*z*) for the range –10 *λ* < z < 10 *λ*. as given by the closed expressions

$$E(0,z)=1-(1+\frac{z}{W})\cos[k(W-z)]+\frac{1}{4}{\left(1+\frac{z}{W}\right)}^{2},$$ (8a)

$$\hat{E}(0,z)=\frac{1}{4}{\left(1-\frac{z}{W}\right)}^{2},\;W=\sqrt{w^{2}-z^{2}}$$ (8b)

which are valid for positive and negative values of *z* and follow readily from Eqs. (9a,b) of Ref. [1] and Eqs. (7a,b), above. In Fig. 2a, the modification of the incident geometrical field is evidenced by the onset of pronounced oscillations of the forward irradiance *E* for *z* < 0. In Fig. 2b, the small but finite values of *Ê* for *z* < 0 demonstrate again that the energy flow is bidirectional and the reverse field reaches into the positive near zone.

It will be noticed that the reverse axial irradiance *Ê*(0,*z*) in Fig. 2b exhibits no oscillations with respect to *z*. This is due to the fact, illustrated in Figs. 3a and b, that *Ê*(*x*,*z*) always has a maximum for *x* = 0. On the source side, this maximum lies in the reflection shadow and is much smaller than the main diffraction pattern formed in the region *x*/*w* > 1.

## 3. Transmission Coefficients

The transmission coefficient *τ* of a diffracting aperture is defined as the radiant flux transmitted into the positive half space, divided by the radiant flux incident upon it in the limit of geometrical optics. Thus, for a plane aperture of area *A* and normally incident parallel light of unit irradiance,

$$\tau =\frac{1}{A}\int_{A}\text{dQ}\;E(Q),$$ (9a)

and for two-dimensional apertures of width 2 *w* which are centered on the coordinate origin, as discussed in this paper, this is further reduced to

$$\tau =\frac{1}{2w}\int_{0}^{w}d\xi \;\text{|}u_{K}\text{(}\xi {\text{,0)|}}^{2},$$ (9b)

where Eq. (3b) was used and the integral over *η* was evaluated as 2 *w*. According to Eq. (3a) one finds

$$\tau =\frac{1}{4w}\int_{0}^{w}d\xi \;{\left|\underset{z\to 0}{\lim}[u_{\text{RS}}^{\left(p\right)}\text{(}\xi ,z)]+1\right|}^{2},\;z>0.$$ (9c)

Here, the aperture value of $u_{\text{RS}}^{\left(s\right)}$ was substituted from Eq. (6d) in order to avoid computational problems that would otherwise arise from singularities for very small values of *z*. The computation of $u_{\text{RS}}^{\left(p\right)}$ involves lesser singularities and could be performed reliably down to *z* = 0.0003 *λ*. Trial computations indicated that the limiting value of *τ* defined by Eq. (9c) was reached at the 0.1 % level for *z* < 0.003 *λ*, and consequently the results presented below were computed for *z* = 0.001 *λ*.

**Fig. 4:**
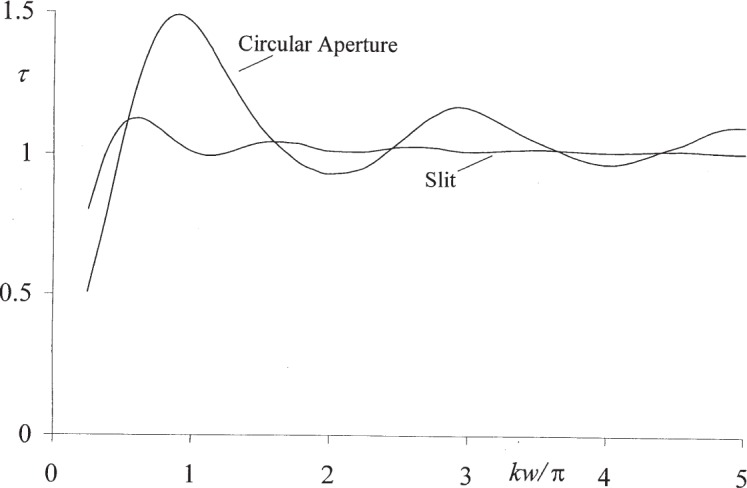
Transmission coefficients of *τ* circular apertures and slits vs *kw*.

The numerical results thus obtained for the transmission coefficients of circular apertures and slits are shown in Fig. 4 for the range 0 < *kw* ≤ 5 π. In both cases, these transmission coefficients approach the limit *τ* = 0 for *kw* = 0, and for larger values of *kw* they exhibit a damped oscillatory behavior. In the case of circular apertures the extremes of *τ* occur near *kw* = π, 2 π, …, whereas for slits they are less pronounced and occur near *kw* = 0.55 π, 1.1 π, …For large values of *kw* outside the range shown in Fig. 4 both approach the limit *τ* = 1, and further computations showed that near *kw* = 100 π the oscillations of *τ* are still on the order of 1 % for circular apertures and less than 0.1 % for slits.

These results can be compared to a large number of data that have appeared in the earlier literature for the limiting case of very small apertures, *kw* → 0. In this limit the transmission coefficients shown in Fig. 4 for circular apertures are superficially similar to, but not the same as those computed by Levine and Schwinger [9,10]. In the case of narrow slits the results obtained here do not at all agree with those published by Bouwkamp [7]. These discrepancies will be addressed in a subsequent publication.

## 4. Concluding Remarks

There are two aspects of the modified theory presented in Sec. 2 that deserve further comments: its failure to account for polarization effects and the appearance of Kirchhoff’s integral in the context of a theory in which metallic screens are assumed.

This work was begun in the anticipation that, because of their pseudo-vectorial nature, the Rayleigh-Sommerfeld integrals could be used to analyze the polarization of diffracted light. This anticipation did not materialize. For example, the expressions derived in Ref. [1] for the axial values of $u_{\text{RS}}^{\left(p\right)}$ and $u_{\text{RS}}^{\left(s\right)}$ pertaining to a circular aperture illuminated by normally incident light differed from each other, although in this case symmetry would dictate the absence of polarization effects. It also seemed odd that the computed values of $u_{\text{RS}}^{\left(p\right)}$ and $u_{\text{RS}}^{\left(s\right)}$ were consistently different in the near zone but the same in the mid zone, without any indication how the degree of polarization could change during the free-space propagation of light. Thus it appeared that, in spite of the assumption of different boundary conditions for $u_{\text{RS}}^{\left(p\right)}$ and $u_{\text{RS}}^{\left(s\right)}$, the “polarization” effects predicted by the Rayleigh-Sommerfeld integrals were implausible.

Whereas the analysis of polarization effects was clearly an objective of Rayleigh’s work [5,6], there is no indication in Sommerfeld’s writings that he had the same goal. In his derivation of Eqs. (2a,b) in Ref. [4], he did not mention polarization at all but stated that separate wave functions and boundary conditions were required to overcome a well-known mathematical inconsistency of Kirchhoff’s theory. In his half-plane work [3,4], he took the additional step of expressing the forward and reverse wave functions in terms of the sums and differences of these separate wave functions, thus negating any semblance with a vectorial theory as these expressions would otherwise imply the interference of mutually orthogonal states. Likewise, any association of the Rayleigh-Sommerfeld integrals with polarized light is negated in the present paper by the introduction of Eqs. (3a) in Sec. 1. Accordingly, the appearance of Kirchhoff’s integral in the modified theory has no significance apart from the fact that it happens to be the arithmetic mean of $u_{\text{RS}}^{\left(p\right)}$ and $u_{\text{RS}}^{\left(s\right)}$.

Given the fact that the modified theory no longer pertains to polarized light, a question arises whether it is still limited to metallic screens. It may be observed that the Eqs. (6b) and (7a) for the forward field are in no way altered if the corresponding expressions for the reverse field are simply ignored, as if the screen were “black.” Thus, these expressions might also be useful to describe the forward field *ν* produced by a black screen, and similarly it might be possible to describe the diffraction by partially reflecting screens by simply multiplying the reverse field $\hat{\nu }$ by a suitable amplitude reflectance. These ideas are akin to earlier suggestions to define blackness as the absence of reflection [11], but cannot be justified theoretically as a metallic screen was assumed in the first place. However, from a pragmatic point of view it appears that the results obtained in this manner would not be far off, and in this context we recall Sommerfeld’s comment [4] that “*a slit scratched in a piece of tin foil produces the same diffraction pattern, no matter if it is shiny or has been blackened.*”

## Footnotes

1 See comments in Sec. 4, below.

2 That is, continuous with continuous first derivatives. The purpose of the derivations in this paper is to make the overall field continuously differentiable with respect to *z*. Continuity with respect to *x* and *y* is assured as the diffracted components of the field will be expressed in terms of Eqs. (2a–c) and obey the wave equation.

3 Because of the two-dimensional nature of the diffraction patterns discussed in the remainder of this paper, the *y*-coordinate will be omitted from here on.
